# HZSM-5 zeolite modification and catalytic reaction mechanism in the reaction of cyclohexene hydration†

**DOI:** 10.1039/d2ra04285a

**Published:** 2022-08-31

**Authors:** Hui Tian, Shuai Liu, Qing Liu

**Affiliations:** College of Chemistry and Chemical Engineering, Yantai University Yantai 264005 China tianhui@ytu.edu.cn

## Abstract

This study investigated a three-phase (liquid–liquid–solid) reaction system of cyclohexene hydration where the catalyst was hydrophilic at the bottom of the water phase. Cyclohexene conversion was low since it was difficult for it to come into contact with the oil. The HCl-OTS-HZSM-5 catalyst was prepared by acid and alkylation modification, then subsequently characterized. Acid modification enabled HZSM-5 zeolite to remove some aluminum atoms, increasing specific surface area, pore volume, and acid sites. *N*-octyltrimethoxysilane (OTS) was grafted onto the HZSM-5 zeolite surface, *i.e.*, alkylation modification, to improve the contact area between immiscible reactants. Consequently, cyclohexene conversion reached 24.07%. Cyclohexene hydration was calculated using the 34T cluster model, and bridging hydroxyl and water molecule adsorption sites were explored. Simulations confirmed that the reaction energy barrier for the HCl-OTS-HZSM-5 catalyzed hydration reaction = 46.67 kJ mol^−1^, considerably less than that with HZSM-5 zeolite (73.78 kJ mol^−1^). The theoretical results reasonably explain the experiments and provide guidance to prepare catalysts with high catalytic activity.

## Abstract 2

This study investigated a three-phase (liquid–liquid–solid) reaction system of cyclohexene hydration where the catalyst was hydrophilic at the bottom of the water phase.

## Graphical abstract

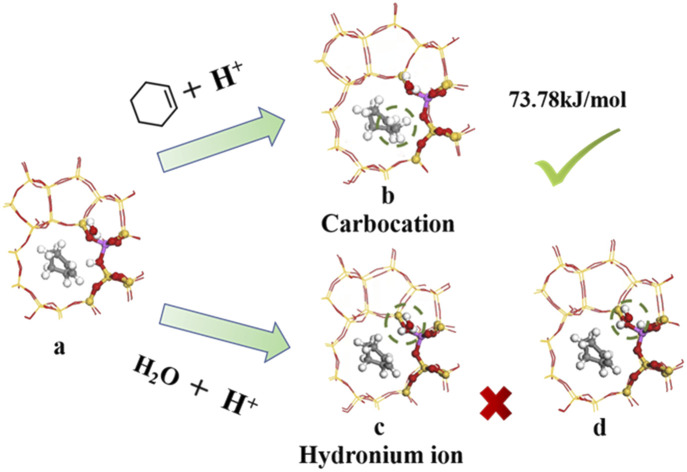

## 1 Introduction

The Si/Al ratio is particularly critical to the catalyst acidity. The presence of aluminum atoms creates a negative charge in the framework that is compensated by hydrogen ions.^1,2^ Hydrogen protons generate strong electrostatic fields enabling strong interactions with polar molecules, but zeolites’ inherent hydrophilicity limits their application.^3–5^

Zeolite alkylation modification by organic alkylation reagents can substantially increase catalyst hydrophobicity.^6^ Adsorption of organic groups on HZSM-5 zeolites can improve hydrophobicity, but also significantly reduces specific surface area and pore volume. The alkylating agent adsorbs onto the zeolite surface to form a multi-layer porous material, blocking catalyst pores. Li *et al.*^7^ modified HZSM-5 zeolite by CLD salinization to not only passivate external surface acidity, but also reduce pore size. Increased active sites can significantly improve olefin conversion efficiency, but this was hindered for alkylation modified microporous zeolite materials.^8^

Previous studies have shown that acid modification can lead to pore expansion. Chen *et al.*^9^ modified zeolite by HCl, increasing specific surface area and pore volume, by reducing silanol groups, and hence reduced zeolite surface affinity for water. Acid modification could improve pore structure and provide more framework acid sites, providing favorable conditions for molecular reactions.^10,11^

HZSM-5 zeolite has excellent catalytic performance for several reactions, including isomerization, alkylation, and aromatization. It also exhibits enhanced aromatic selectivity and organic liquid product yield compared with other catalysts.^12,13^ Density functional theory (DFT) has been widely used to study factors affecting catalytic reaction mechanisms, including adsorption configuration, Gibbs reaction energy barrier, reaction transition state, *etc.*^14–21^ Fu *et al.*^22^ used DFT for 8T and 48T models with the mGGA-M06-L function to study mono-branched alkanes in HY and HZSM-5 adsorption energies. They concluded that pore confinement was critical to adsorbate stability. Rosenbach^23^ and Mullen^24^ showed that carbocations occurred in the zeolite channel in the alkoxy group configuration using HZSM-5 catalyst. Carbocation was confined to near the oxygen atom, and the central carbon atom was connected with the oxygen atom on the zeolite framework by covalent bonds.^25,26^

MFI zeolite has been widely to study catalysis. This paper modified HZSM-5 zeolite by acid and alkylation to obtain HCl-OTS-HZSM-5 zeolite catalyst, then subsequently investigated catalyst acid sites and hydrophobic properties to explore optimal cyclohexene conversion. The catalytic cracking mechanism for HZSM-5 zeolite was investigated using DFT, calculating elementary reaction Gibbs energy barriers through simulation. The theoretical calculation results reasonably explain experimental outcomes and provide guidance to prepare of catalysts with high catalytic activity.

## 2 Materials and methods

### 2.1 Catalyst preparation

HZSM-5 zeolite was obtained from Nankai University, China. We chose HZSM-5 zeolite with Si/Al = 25 because it offered high acid site density to better promote cyclohexene hydration. (Octyl)-trimethoxysilane (OTS) was obtained from MACKLIN. Methylbenzene, ethyl alcohol, and carbon tetrachloride were provided by SINOPHARM. HCl acid was obtained from Yantai San he Chemical Reagent Co., Ltd. The specific preparation steps have been described in previous work.^27,28^ The best HCl-HZSM-5 was selected for 25% alkylation modification^27^ (hydrochloric acid modification concentration = 4 mol L^−1^).^28^ The HCl-OTS-HZSM-5 catalyst was prepared by acid and alkylation modification.

### 2.2 Catalyst evaluation

The reactants were added to the reaction kettle for reaction, and the products were separated for quantitative analysis by gas chromatography after reaction. The main product was cyclohexanol, which the by-products were small amounts of methylcyclopentene and methylcyclopentanol. The calculation formulas of cyclohexene conversion and cyclohexanol selectivity were as follows:1
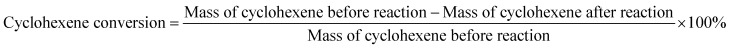
2
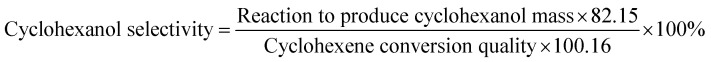


**Fig. 1:**
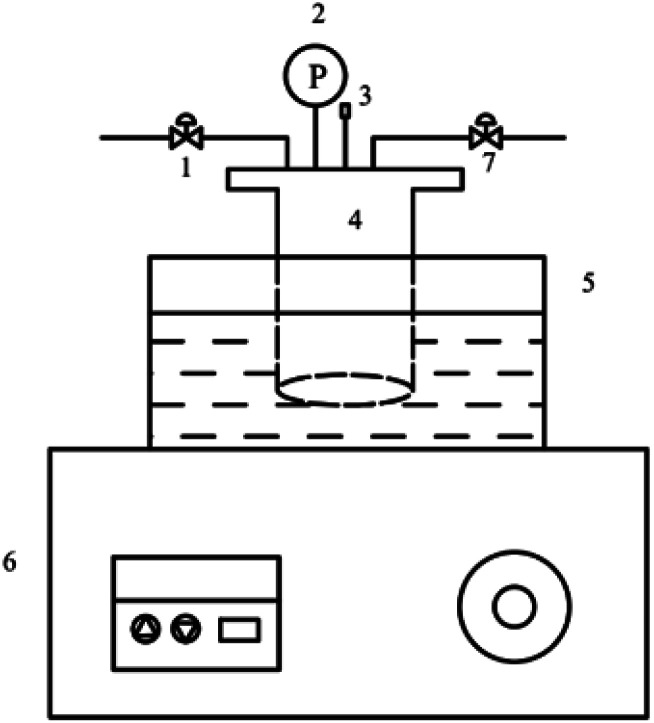
Diagram of cyclohexene hydration reaction device. 1. Inlet valve. 2. Pressure gauge. 3. Thermocouple. 4. Reactor. 5. Oil bath. 6. Temperature display controller. 7. Outlet valve.

The catalyst evaluation device was shown in Fig. 1. The reactants were added to the autoclave and replaced with nitrogen for 3–5 times. After the temperature of the reaction kettle rises to a certain temperature, the timing was started, and the reaction was completed for a period of time. The reaction kettle was immediately quenched in a cold-water bath, and the reaction solution was poured into a centrifuge tube. Solid–liquid separation was performed by centrifugation, and the catalyst was recovered. The water–oil biphasic product was separated using a separating funnel, and the aqueous phase was extracted with 1,2-dichloroethane. The obtained extract phase and oil phase were added with internal standard ethanol, respectively, and analyzed by gas chromatography.

### 2.3 HZSM-5 model

**Fig. 2:**
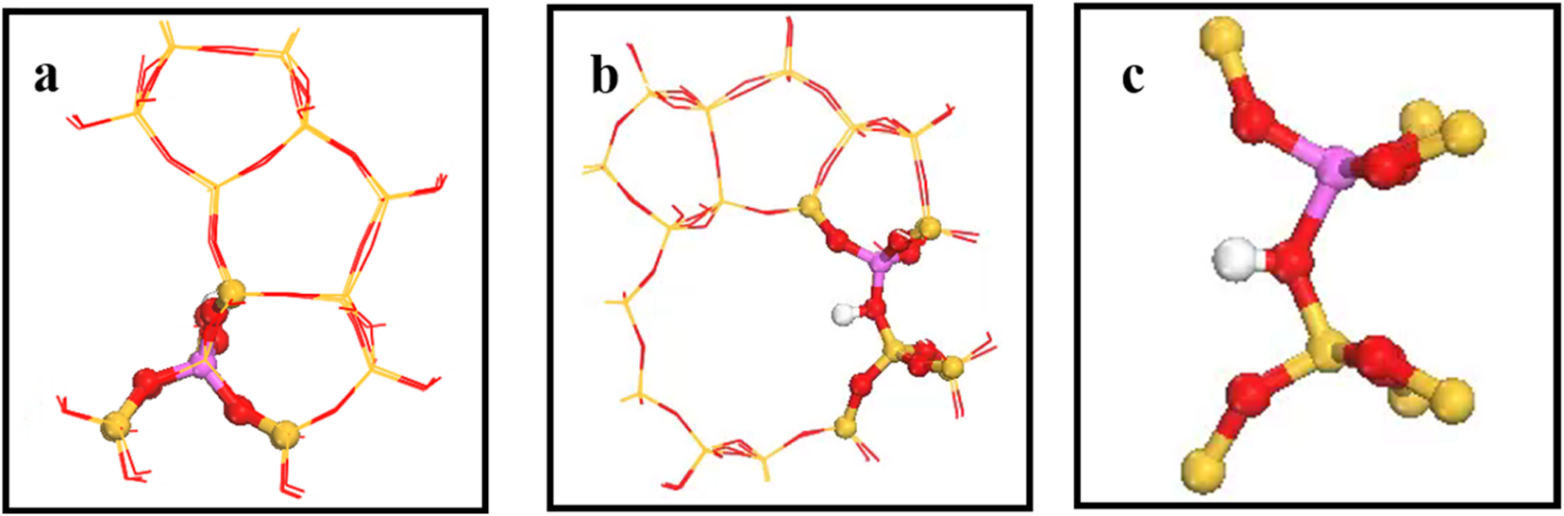
a) and (b) are HZSM-5 zeolite 34T cluster models: O atoms are red, Si atoms are yellow, H atoms are white, and the aluminum atom at the T12 position is purple. (c) Is 8T structure.

Fig. 2 shows the 34T cluster model employed to represent the HZSM-5 zeolite, following previous studies.^29^ ZSM-5 zeolite comprised two intersecting 10-ring channels forming sinusoidal channels moving along the crystal *a*-axis and straight channels along the crystal *b*-axis, with corresponding 5.5 × 5.1 and 5.3 × 5.6 Å pore sizes, respectively. The 10-ring channel comprised 10 T atoms, where T represents Si or Al. Replacing T12(Si) with Al reduced steric hindrance for larger intermediates in the framework (see Fig. 2(a) and (b)). Artificially cutting the Si–O bond caused the boundary atoms to produce unrealistic dangling bonds, with consequential boundary effects. The boundary effect was reduced by adding hydrogen atoms since H and Si have similar same electrical properties and the Si–H bond direction was consistent with the Si–O bond direction in the periodic model. Replacing a silicon atom by an aluminum atom cased the zeolite framework to become negatively charged, which was added to the zeolite by introducing hydrogen protons to maintain the structure’s electroneutrality, and also forming a Brønsted acid site.^30^Fig. 2(c) shows the (SiO)_3_–Si–OH–Al–(OSi)_3_ 8T structure was relaxed to reduce the calculation overhead and prevent the structure from collapsing during geometry optimization, and the remaining part was fixed on crystal coordinates.

## 3 Catalyst results and discussion

### 3.1 HCl-OTS-HZSM-5 zeolite characterization

#### 3.1.1 XRD

**Fig. 3:**
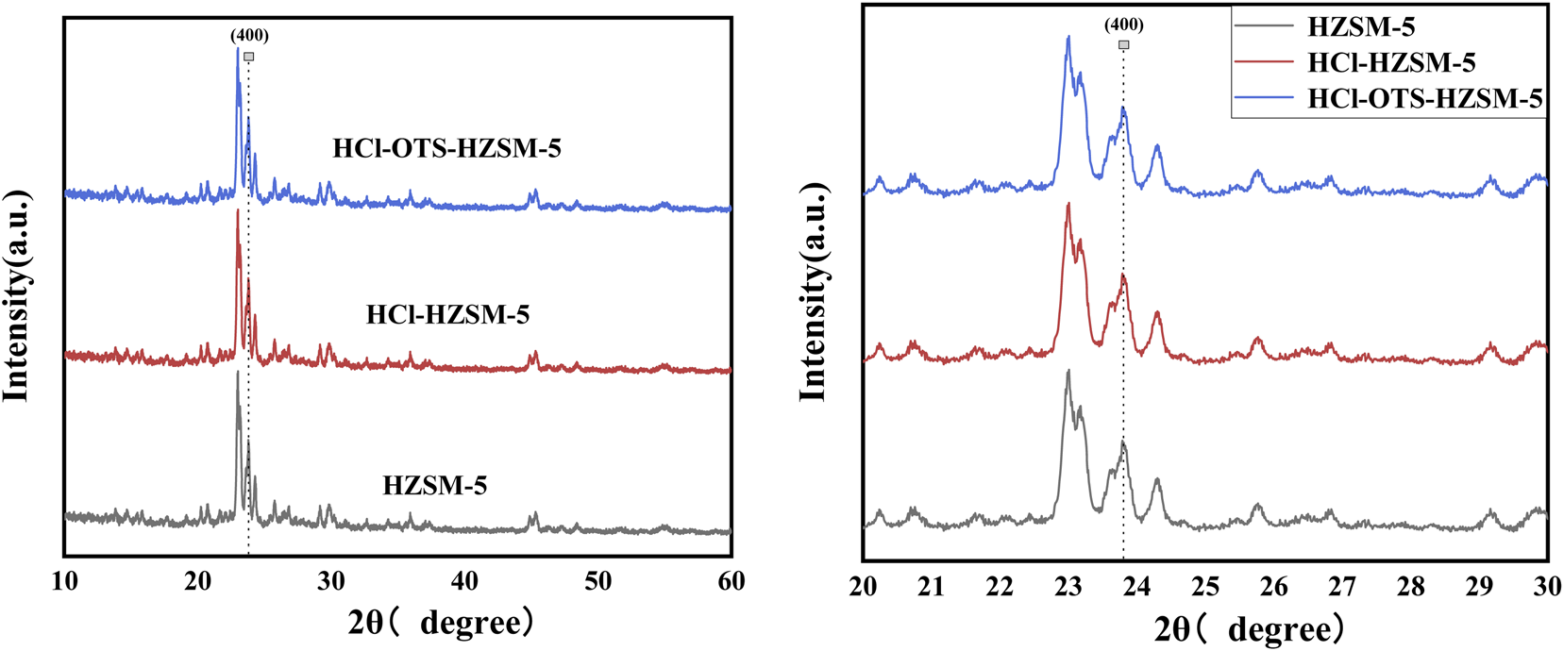
XRD patterns of HZSM-5, HCl-HZSM-5 and HCl-OTS-HZSM-5 zeolites.

Fig. 3 shows typical XRD patterns for HZSM-5, HCl-HZSM-5, and HCl-OTS-HZSM-5 exhibit characteristic MFI zeolite diffraction peaks at 23–30°. The crystal was reduced at (400) for HCl-HZSM-5 and HCl-OTS-HZSM-5, mainly due to removing some Al atoms after acid modification, hence reducing the crystal. No characteristic OTS peaks occurred for the HCl-OTS-HZSM-5 zeolite, indicated that alkylation modification did not change the crystal structure. Thus, deposited alkylating agent (OTS) was highly dispersed, *i.e.*, amorphous phase, during alkylation modification.

#### 3.1.2 FT-IR

**Fig. 4:**
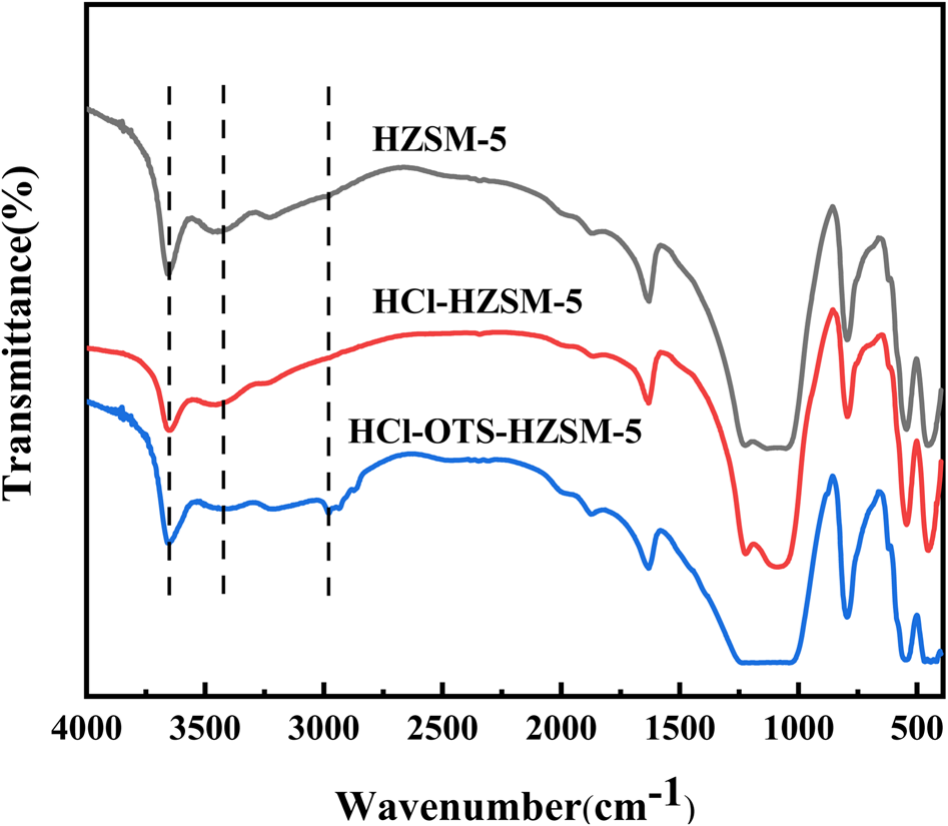
FT-IR spectra of HZSM-5, HCl-HZSM-5 and HCl-OTS-HZSM-5 zeolites.

Fig. 4 shows FT-IR spectra for HZSM-5, HCl-HZSM-5, and HCl-OTS-HZSM-5 exhibit characteristic absorption near 450, 550, 840, 1095, 1220 and 1633 cm^−1^ peaks, confirming that all three catalysts were MFI-type zeolites. The peak around 3450 cm^−1^ was ascribed to terminal hydroxyl group and hydrogen-bonded adjacent hydroxyl group stretching vibrations.^33^ Absorption peak intensities for the HCl-HZSM-5 and HCl-OTS-HZSM-5 hydroxyl groups reduced compared with HZSM-5. Surface wettability could have changed from hydrophilic to hydrophobic and the modified catalyst characteristic peak at 3610 cm^−1^ intensity decreased due to Al atom removal reducing Si–OH–Al groups. The HCl-OTS-HZSM-5 sample exhibits an infrared absorption peak ∼3000 cm^−1^, mainly due to C–H stretching vibration in the CH_3_-group. The OTS C8 hydrophobic chain was successfully adsorbed on the zeolite surfaces, forming a densely packed alkyl chain layer.

#### 3.1.3 TPD and pyridine-IR

**Fig. 5:**
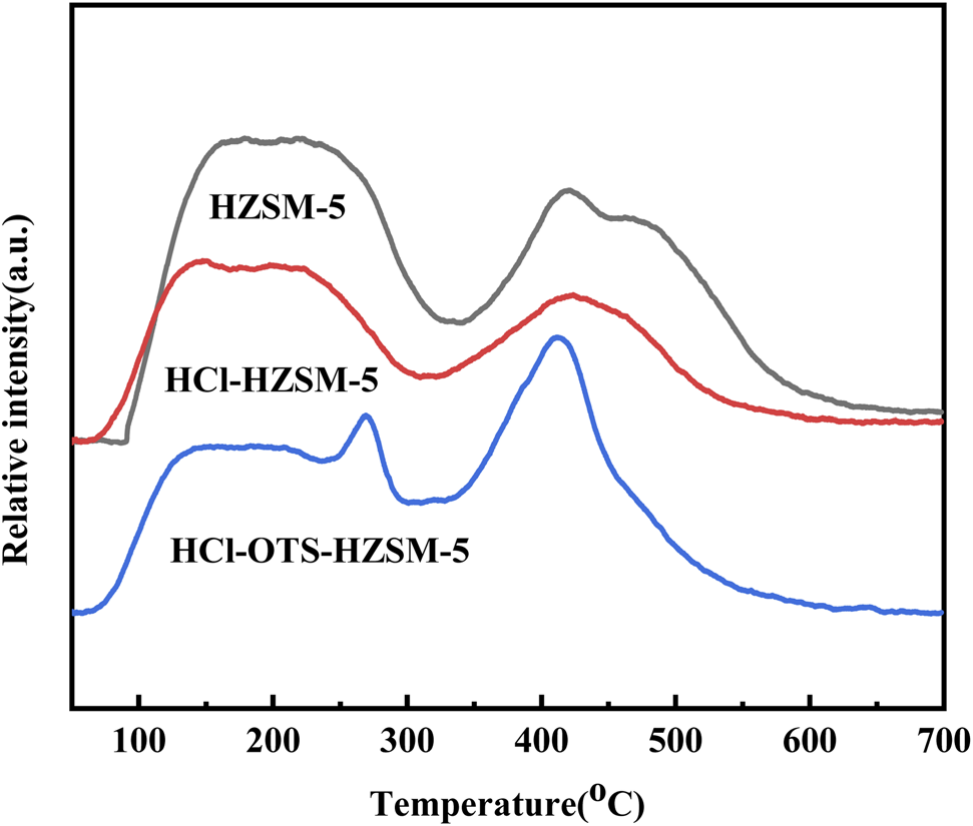
TPD profile of HZSM-5, HCl-HZSM-5 and HCl-OTS-HZSM-5 zeolites.

Fig. 5 shows NH_3_-TPD (Ammonia adsorption curve) for HZSM-5, HCl-HZSM-5, and HCl-OTS-HZSM-5 all exhibit two absorption peaks: a weak acid center in the low temperature region (about 150 °C) and strong acid center in the high temperature region (about 400 °C). The low temperature peak was mainly due to the weak acid center for the external skeleton Al, and the high temperature peak mainly due to the strong acid center for the internal skeleton Al.^34^ The weak acid center peak shifted forward after acid modification. It was difficult for OTS molecules to enter zeolite channel interiors due to zeolite’s relatively small size; but they covered the outer surface, hence covering acid sites on the outer surface.

**Fig. 6:**
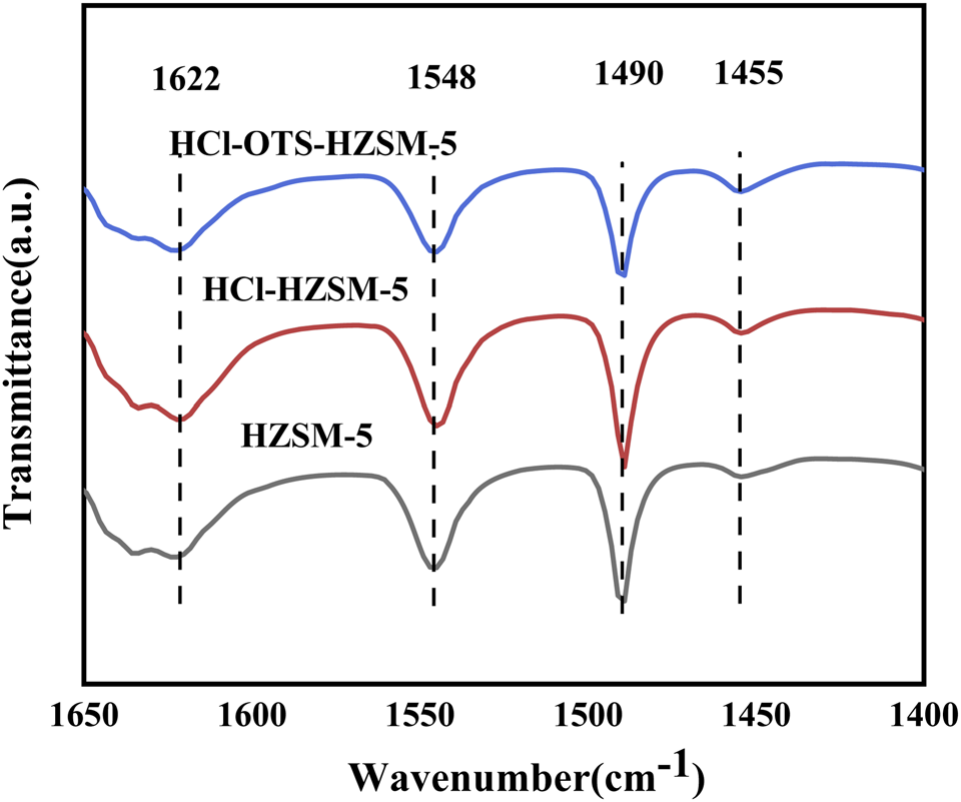
Pyridine-IR spectra of HZSM-5, HCl-HZSM-5 and HCl-OTS-HZSM-5 zeolites at 200 °C and atmospheric pressure.

Fig. 6 and Table 1 show zeolite B and L acid regions distinguished by Py-IR adsorption infrared spectroscopy. B and L acid centers occurred in all samples, with B acid content decreasing slightly after acid modification, from 348 to 338 mmol g^−1^; whereas L acid content decreased significantly, from 41 to 33 mmol g^−1^. This was mainly due weak Al acidity, hence removing Al reduced weak acid content, and retained B acid enhanced olefin hydration. In contrast, the OTS-HCl-HZSM-5 zeolite exhibits considerably different strong and weak acid reductions, mainly because OTS was grafted on the catalyst surface, covering some acid sites and hence reducing acid content. Physical OTS adsorption bridged hydroxyl groups, causing pore blockages and hence reducing Brønsted and Lewis acid sites.

**Table 1:** Calculated acid of HZSM-5, HCl-HZSM-5 and HCl-OTS-HZSM-5 zeolites

Sample	Acid area		Acid amount (mmol g^−1^)		Total acid (mmol g^−1^)
	Brønsted Lewis		Brønsted Lewis		
HZSM-5	8.71	1.36	348	41	389
HCl-HZSM-5	8.34	1.07	338	33	371
HCl-OTS-HZSM-5	7.00	1.01	279	31	310

#### 3.1.4 Nitrogen adsorption and desorption

**Fig. 7:**
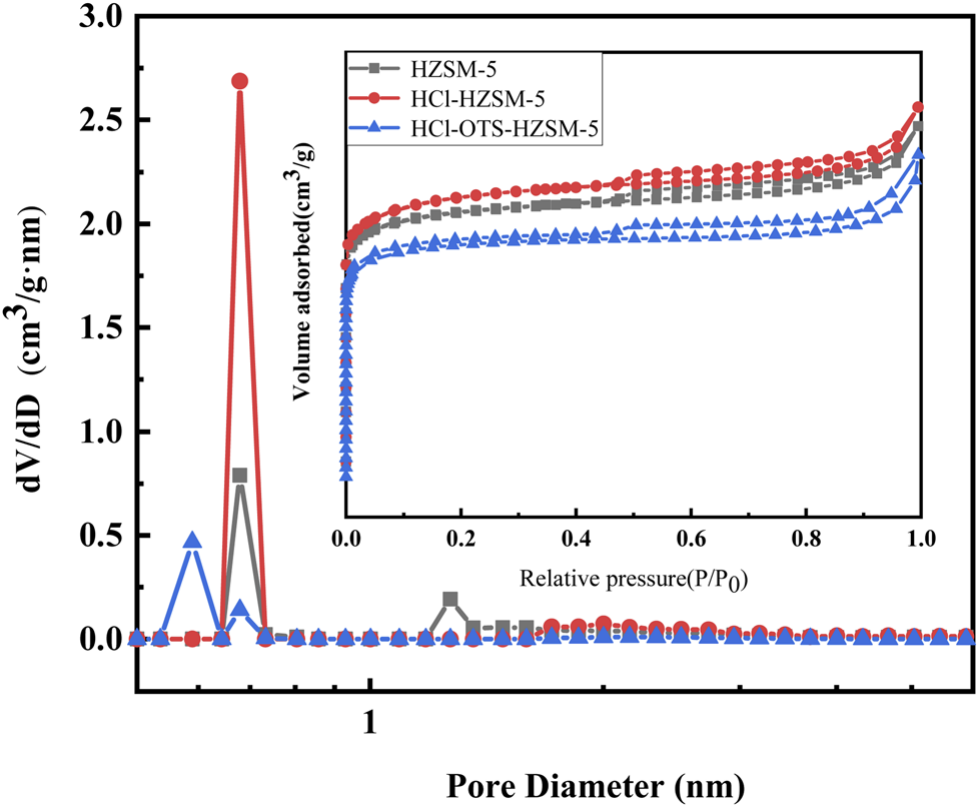
N_2_ adsorption and desorption isotherms and pore diameter distribution of HZSM-5, HCl-HZSM-5 and HCl-OTS-HZSM-5 zeolites (adsorption isotherms for nitrogen at 77 K).

Fig. 7 shows N_2_ adsorption isotherms of all catalyst samples in the IV microporous materials. Table 2 shows structural parameters for the three catalysts. HCl-OTS-HZSM-5 zeolites increased from 264 to 280 m^2^ g^−1^; HCl-OTS HZSM-5 reduced to 232 m^2^ g^−1^, and total pore volume reduced to 0.14 cm^3^ g^−1^. The alkylating agent (OTS) had difficulty entering the zeolite interior and connecting with hydroxyl groups through the “Si–O–Si” bond. Thus, OTS deposited on the outer catalyst surface, forming a thick hydrophobic layer, effectively reducing the porosity and increasing surface resistance to N_2_. The plugged mesoporous structure changed intergranular pore size, hence greatly increasing catalyst specific surface area and pore volume compared with previous studies.^25^

**Table 2:** The BET surface area, pore diameter and pore volume of HZSM-5, HCl-HZSM-5 and HCl-OTS-HZSM-5 zeolites

Sample	*S*_BET_ a (m^2^ g^−1^)	Pore volume (cm^3^ g^−1^)		
		*V*_total_ b	*V*_micro_ c	*V*_meso_ d
HZSM-5	264	0.16	0.11	0.05
HCl-HZSM-5	280	0.17	0.11	0.06
HCl-OTS-HZSM-5	232	0.14	0.11	0.03

a Specific surface area (*S*_BET_), estimated by N_2_ adsorption at 77 K using BET method.

b Total pore volume (*V*_total_), calculated form the adsorption capacity at *P*/*P*_0_ = 0.95.

c Microporous volume (*V*_micro_), determined by *t*-plot method.

d Microporous volume (*V*_meso_), *V*_meso_ = *V*_total_ – *V*_micro_.

#### 3.1.5 Contact angle

**Fig. 8:**
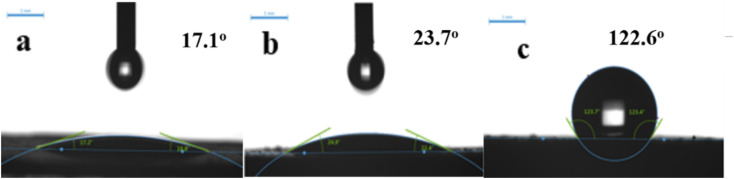
Contact angle variation on (a) HZSM-5, (b) HCl-HZSM-5 and (c) HCl-OTS-HZSM-5 zeolite.

Fig. 8 shows contact angle (CA), to investigate sample wettability. Unmodified samples exhibit good hydrophilicity with CA ≤ 20°. Al atoms were removed from the zeolite framework by acid modification, destroying Al–O bonds and enhancing Si–O bonds. Since the Si–O–Al bond was more polar than Si–O–Si (the covalent bond had higher binding energy), the zeolite became less polar and more hydrophobic. Thus, HCl-OTS-HZSM-5 zeolite CA = 122.6° was modified by alkylation to determine the hydrophobicity. Acid modification enlarges pore volume, exposing more hydroxyl groups and hence OTS molecules became more easily adsorbed on the zeolite surface. Therefore, we achieved significant CA improvement compared with previous studies.^25^ Acid modification increases hydrophobicity by removing aluminum atoms and reducing the polarity of molecular sieves; while alkylation modification increases hydrophobicity by adsorbing C8 hydrophobic chains on the surface of molecular sieves through “Si–O–Si” bonds. From the macroscopic observation, the modification effect of molecular sieve alkylation is more obvious.

#### 3.1.6 TGA

**Fig. 9:**
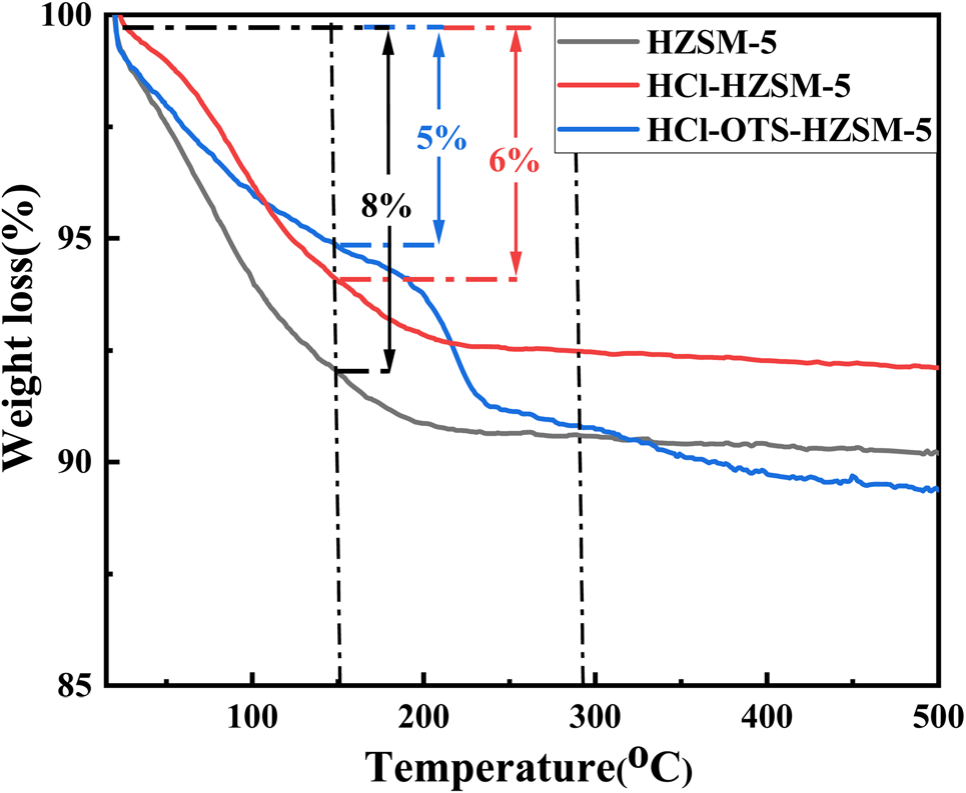
Thermogravimetric curves of HZSM-5, HCl-HZSM-5 and HCl-OTS-HZSM-5 zeolites. Left axis: weight (%).

Fig. 9 shows TGA profiles to assess sample stability. Mass loss from 0 to 150 °C was mainly attributed physiosorbed water removal. The HZSM-5 sample with lower Si/Al ratio adsorbed more water, confirming enhanced hydrophilicity, and adsorbed up to 8 wt% water; whereas HCl-HZSM-5 and OTS-HCl-HZSM-5 samples with higher Si/Al ratio adsorbed only 6 and 5 wt% water, respectively. Thus, acid and alkylation modified samples were more hydrophobic. Catalyst mass for HZSM-5 and HCl-HZSM-5 no longer changed with increasing temperature after physiosorbed water desorption. However, OTS-HCl-HZSM-5 suffered serious mass loss, mainly due to *n*-octyltrimethylsilane (OTS) detachment from the zeolite surface at high temperature.

#### 3.1.7 XPS

**Fig. 10:**
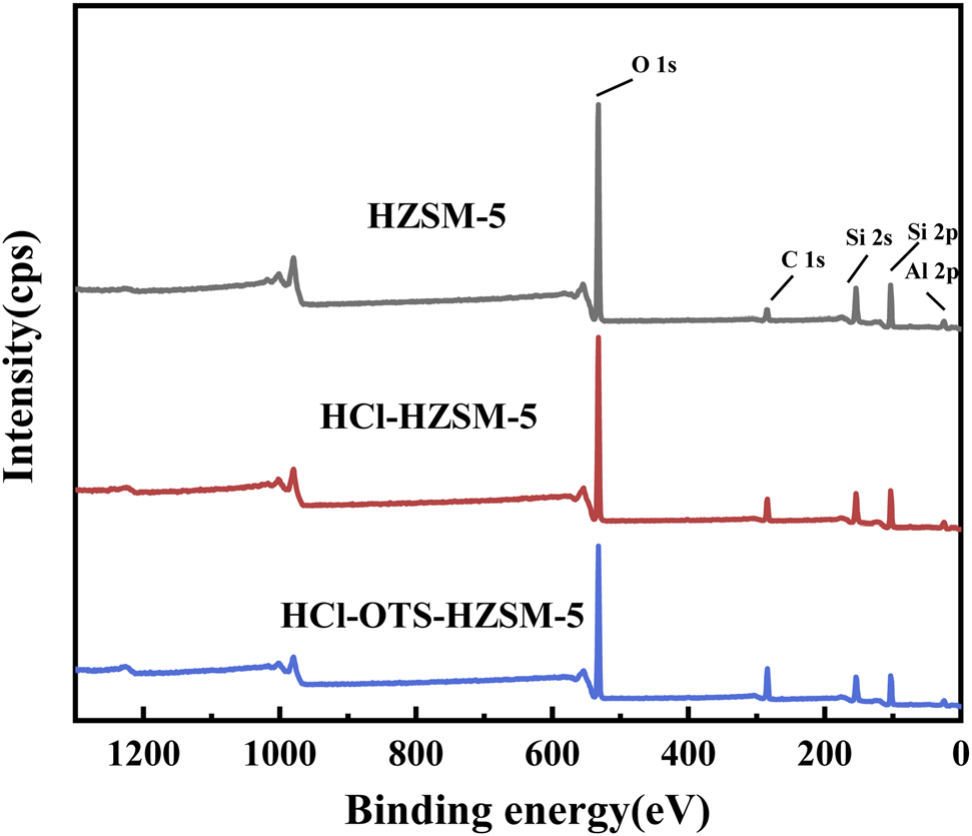
XPS survey scan spectra of HZSM-5, HCl-HZSM-5 and HCl-OTS-HZSM-5 zeolites.

Fig. 10 shows elemental spectra from C1s, O1s, Al2p, Si2s and Si2p XPS profiles over binding energy ranges from 0 to 1250 eV; and Table 3 shows corresponding calculated elemental contents. Acid modification effectively removed Al. Efficient OTS coverage increased carbon signal; whereas effective organosilanes coverage strongly enhanced the carbon signal and decreased both silicon and oxygen signal intensities. Thus, XPS results confirm *n*-octyltrimethoxysilane was covalently bonded to HZSM-5 zeolite surface, consistent with FT-IR outcomes (Fig. 4).

**Table 3:** Calculated surface composition of HZSM-5, HCl-HZSM-5 and HCl-OTS-HZSM-5 zeolites

Samples	Concentration of elements (%)			
	O	Si	C	Al
HZSM-5	60.52	27.76	9.92	1.79
HCl-HZSM-5	60.15	27.93	10.25	1.66
HCl-OTS-HZSM-5	49.03	24.18	25.27	1.51

### 3.2 HCl-OTS-HZSM-5 catalytic activity

Previous studies evaluated catalysts under static conditions, whereas the current study considered dynamic temperature, water-ene molar ratio, reaction time, catalyst dosage, and catalyst recycling.

Forward reaction rate increased and equilibrium shifted toward the forward reaction direction with increasing temperature since olefin hydration is reversible exothermic. Cyclohexanol selectivity decreased significantly as temperature was raised further, probably due to by-products caused by the high temperature. Optimum reaction temperature = 130 °C.

Excess water was selected to participate in the reaction due to high cyclohexene raw material prices. Cyclohexene conversion first increased and then decreased with increasing water molar ratio, whereas selectivity remained above 99%. Optimum water to ene molar ratio = 6. The reaction tends to balance with increasing reaction time, and conversion rate was not significantly improved by extending the reaction time. Thus, optimal reaction time = 4 h.

Cyclohexene hydration was acid-catalyzed, and increasing centers provided stronger catalytic activity. However, the conversion rate did not increase significantly once sufficient catalyst was present, and excess catalyst tended to cause separation difficulty. Thus, optimum catalyst dosage = 1.65 g, *i.e.*, 10 wt% relative to water mass.

**Fig. 11:**
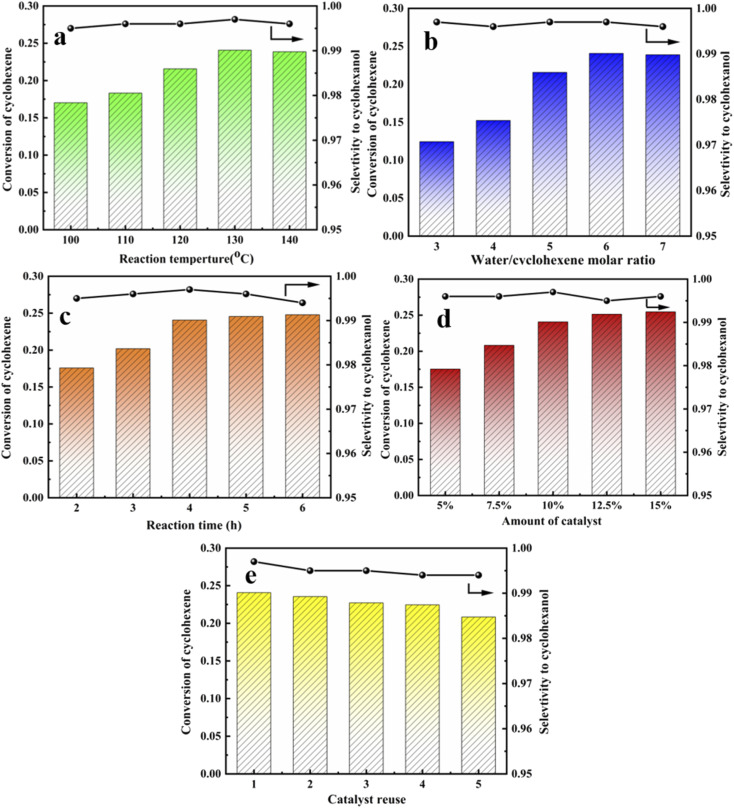
Cyclohexene conversion of the HCl-OTS-HZSM-5 zeolite. (a) The different reaction temperature (100 °C, 110 °C, 120 °C, 130 °C, 140 °C); (b) the different water/cyclohexene molar ratios (3, 4, 5, 6, 7); (c) the different reaction time (2 h, 3 h, 4 h, 5 h, 6 h); (d) the different amount of catalyst (5%, 7.5%, 10%, 12.5%,15%); (e) catalyst recycles (1, 2, 3, 4, 5). Optimum reaction conditions: reaction temperature = 130 °C, water/cyclohexene molar ratios = 6, reaction time = 4 h, catalyst dosage = 10.5%.

Fig. 11 shows repeated catalyst cycling in the reaction kettle. Cyclohexene conversion decreased somewhat after 5 cycles, but cyclohexanol selectivity did not change significantly. This may be because more organic matter adhered to the catalyst surface, covering some acid centers and hence affecting catalyst activity. Thus, catalyst regeneration was required. Organic material was washed away using ethanol and suction filtration, then dried at 120 °C for 12 h to restore catalyst activity. Optimal reaction conditions for cyclohexene hydration were reaction temperature = 130 °C, water to ene molar ratio = 6, reaction time = 4 h, and catalyst dosage = 1.65 g (10 wt% relative to water mass). Maximum conversion rate = 24.07%, and selectivity remained at 99%.

## 4 Simulation

### 4.1 Cyclohexene and water molecule adsorption

We calculated cyclohexene reaction energy barriers and compared with kinetic parameters to verify parameter selections and calculated results rationality. Activation energy for HZSM-5 catalyzed cyclohexene hydration reaction = 77.69 kJ mol^−1^^35^ from estimated kinetic model parameters; whereas simulations predicted energy barrier = 73.78 kJ mol^−1^. Thus, the model and parameter settings were confirmed to be reliable.

**Fig. 12:**
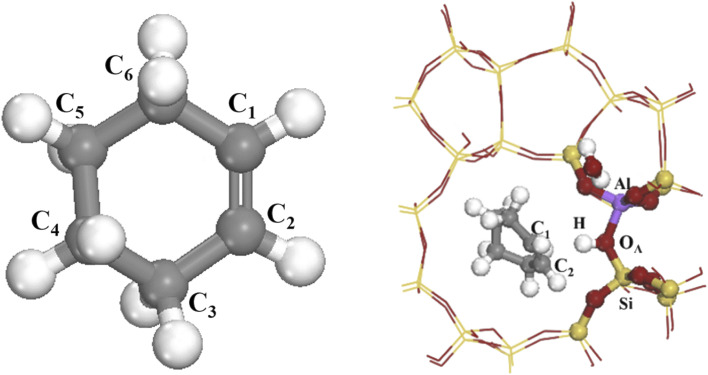
Cyclohexene and optimized geometries of adsorbed molecules on the Brønsted site of the HZSM-5.

Fig. 12 shows stable adsorption configurations for cyclohexene and water molecules on the HZSM-5 cluster model, with corresponding atoms marked. Cyclohexene molecules mainly cluster at straight sinusoidal and sinusoidal channel intersections due to confinement effects from the pores.

Tables S1 and S2† summarize Mulliken electron layouts and atomic distances before and after adsorption. Oxygen atom (O_A_) Mulliken charge on the acidic site reduced from −1.192 to −1.237 eV after olefin molecule adsorption, with H atom charge increasing to 0.616 eV, and O_A_–H bond length increasing from 0.978 to 0.997 Å, due to forming π–H bonds between cyclohexene molecules and HCl-HZSM-5 zeolite. The C_2_ atom on the cyclohexene molecule lost electrons, and hence C_1_–C_2_ bond length increased from 1.331 to 1.498 Å. O_A_–H bond length on the zeolite increased from 0.978 to 0.997 Å, and both Al–O_A_ and Si–O_A_ bond lengths shortened due to the C=C double bond influence; and H^+^ tended to be protonated at the acidic site. Some electrons transferred from hydrogen atoms to the double-bonded carbon atoms, hence double-bonded C atom electron density increased and O_A_–H bond length increased. A hydrogen bond formed between the cyclohexene molecule and bridging hydroxyl group, allowing electron flow from the cyclohexene molecule to the zeolite model, increasing cyclohexene molecule positive charge.^36^

### 4.2 Electron density distribution and LUMO and HOMO

**Fig. 13:**
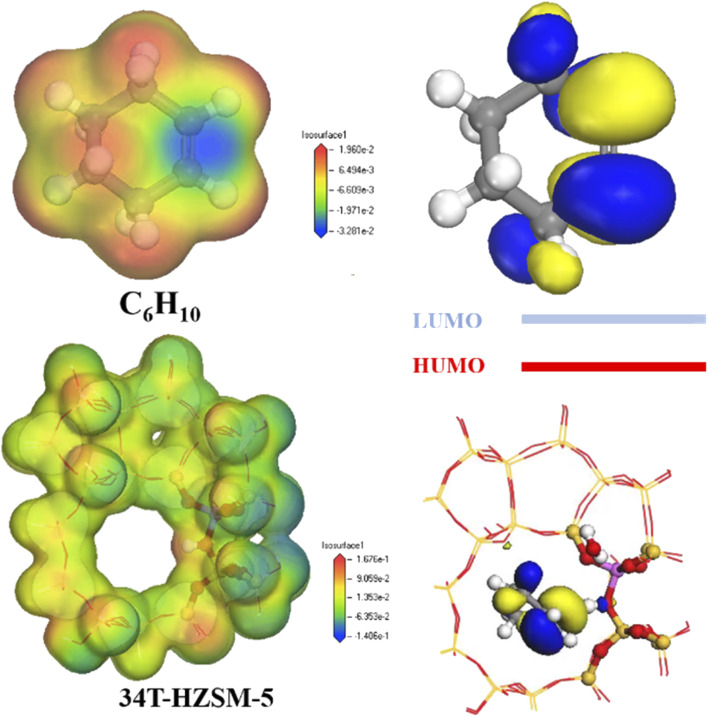
Electron density distribution and LUMO and HOMO orbitals.

Fig. 13 shows electron density for cyclohexene and zeolites. Electron density was lowest at the carbon–carbon double bond for cyclohexene, and highest at the hydrogen proton for zeolite.

The cyclohexene molecule will be repelled due to electrostatic repulsion when its high electron density region was close to the zeolite framework. Stabilizing effects from channel confinement were also weak at this time, hence the zeolite framework separated from the adsorbate. Only there was a certain distance between the zeolite framework and the adsorbate, the carbon–carbon double bond of cyclohexene was close to the hydrogen proton. The low electron density region occupied the channel, and the stabilization effect produced by the channel confinement effect was the strongest. This made the smaller pore size HZSM-5 zeolite, which could stabilize the cyclohexene molecules in the pores by electrostatic action.^37^

Highest occupied orbital (HOMO) and lowest unoccupied orbital (LUMO) for the reactant molecule are key to determining the chemical reaction system. Zeolite catalyst HOMO orbitals were mainly distributed on the oxygen atoms adjacent to the Al phase, whereas cyclohexene LUMO orbitals were mainly distributed on the double-bonded carbon atoms (Fig. 12). Thus, catalyst hydrogen proton HOMO matched cyclohexane LUMO symmetry, enabling electron transfer from the HOMO to symmetry-matched LUMO orbitals (see number of electrons in Table 3). Therefore, high energy HOMO orbitals in the zeolite channel indicates strong electron donation in the reaction, and H^+^ was preferentially transferred to the double-bonded carbon atoms.

### 4.3 Molecular adsorption site

#### 4.3.1 Bridging hydroxyl protonation sites

**Fig. 14:**
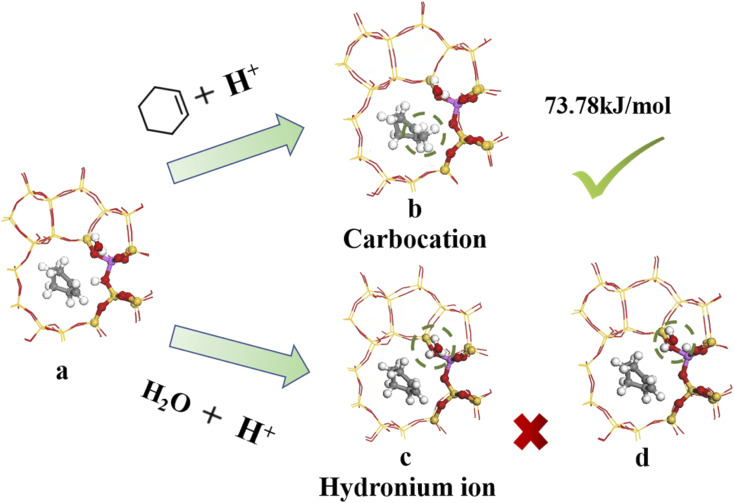
Bridged hydroxyl protonation site.

Fig. 14 shows the bridged hydroxyl group protonation position comprises two reaction paths: cyclohexene protonation produced carbocations, and water molecule protonation formed hydronium ions.

Fig. 14(a) and (b) show reaction product and optimized product stability, with calculated reaction energy barrier = 73.78 kJ mol^−1^. The transition state search was performed on the reactants and produced to explore the highest reaction energy barrier.

Fig. 14(c) shows optimal hydronium ion reaction production, decomposing hydronium ions into water molecules and hydrogen ions once the reaction completes, and transferring hydrogen ions to oxygen atoms to form bridging hydroxyl groups, as shown in Fig. 14(d). Thus, hydronium ions were unstable when alkenes occurred and hydrogen protons transferred to the zeolite framework to reform B acid sites. Joshi *et al.*^38^ confirmed hydronium ion instability under low water loading by RMD simulation, showing difficulty to form water clusters, hence proton transfer stalled and the protons eventually returned to the zeolite catalyst, retaining zeolite acidity. Liu *et al.*^39^ showed that hydronium ions exhibited higher barrier to alkene protonation than bridging hydroxyl protonation, which preferentially underwent bridging hydroxyl protonation. Thus, bridging hydroxyl groups preferentially protonated the alkene during cyclohexene hydration at low water loading (Fig. 14(a) and (b)).

#### 4.3.2 Water molecule adsorption sites

**Fig. 15:**
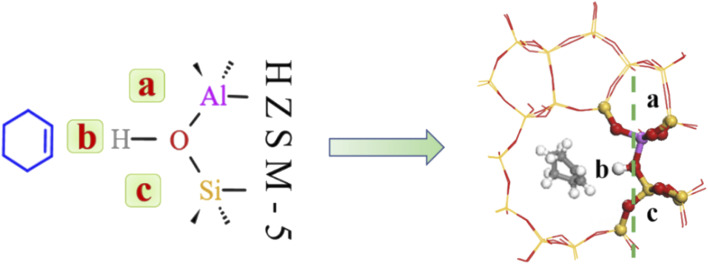
Water adsorption site.

Bridging hydroxyl groups in the zeolite framework were strongly acidic and protons (H^+^) were exchanged on adjacent oxygen atoms in HZSM-5 to maintain charge neutrality.^40,41^ Water molecules are strongly adsorbed near these acidic centers to form protonated water clusters at higher water loadings; whereas water molecules formed hydrogen-bonds with HCl-HZSM-5 zeolite at low water loading. Fig. 15 shows that the reaction region can be divided into a, b, c and d regions due to the presence of water molecules.

**Fig. 16:**
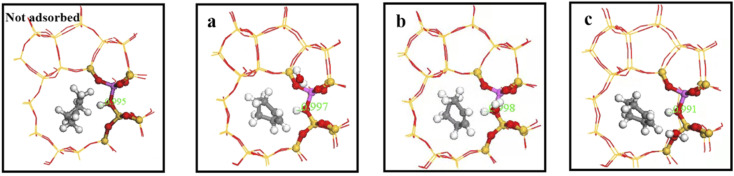
O_A_–H bond lengths of water molecules in a, b and c regions.

Consequently, we investigated interaction between water molecules and Si–OH–Al sites during hydration by adding a single water molecule to identified regions, and calculating O_A_–H bond lengths, as shown in Fig. 16. Distance between framework oxygen and hydrogen atoms = 0.995 Å in the absence of adsorbed water molecules; whereas O_A_–H bond lengths = 0.997, 0.998, and 0.991 in the a, b, and c regions, respectively, when water molecules were adsorbed on the zeolite framework.

Acidic zeolite catalytic sites changed with the presence of water molecules, and changing O_A_–H bond length changed Brønsted acid site properties. Bridging hydroxyl groups were stable under low water loading, and O_A_–H bond lengths lengthened in the a and b regions due to O_A_–H bond interaction with hydrogen bonding of water molecules. Rapid proton exchange between bridging hydroxyl groups and water molecules occurred when water molecules were in the a and b regions, increasing zeolite acidity. However O_A_–H distance and proton mobility did increase significantly when a single water molecule was in the c region.

**Fig. 17:**
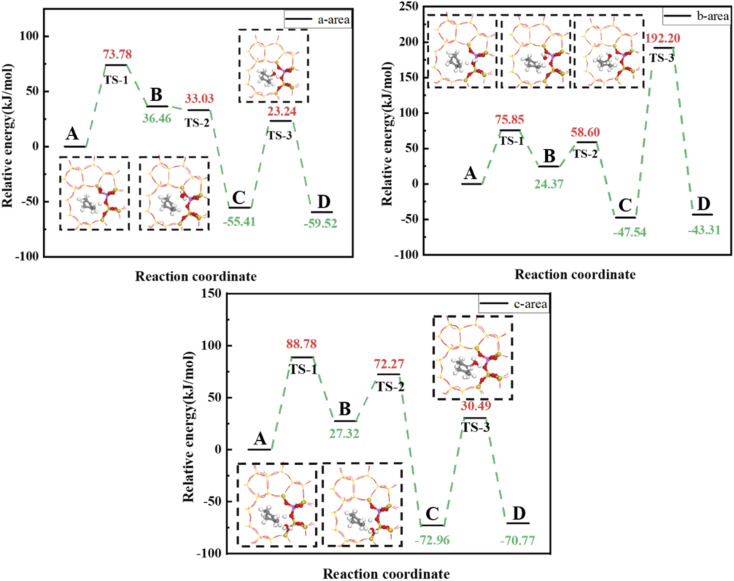
Energy barriers for cyclohexene hydration catalyzed by HZSM-5 in a, b and c regions.

**Fig. 18:**
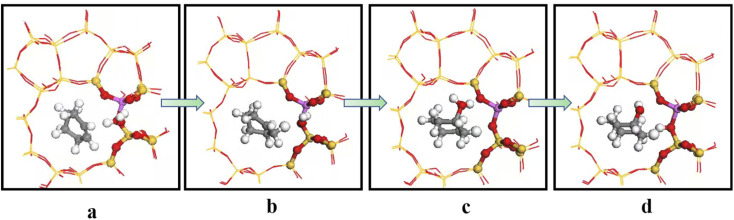
Schematic diagram of adsorption in region b.

Fig. 17 shows calculated energy barriers for cyclohexene hydration reactions with water molecules in the three regions >88.78, 75.85, and 73.78 kJ mol^−1^ for regions c, b, and a, respectively. This ordering may be due to O_A_–H bond length was only 0.991 Å in the c region, and weak B acid strength made proton transfer difficult. Thus, the first step energy barrier for acid catalysis reaction was high (88.78 kJ mol^−1^, Fig. 17(c)). The reaction energy barrier for dehydrogenation in the b region was as high as 192.20 kJ mol^−1^, which may be due to steric hindrance making dehydrogenation difficult, as shown in Fig. 18(c) and (d). Wang *et al.*^18^ showed that ^29^Si resonance in analyzed ^29^SiNMR spectra produced larger downfield shift when interacting with protons, resulting in less shielding relative to trivalent Al ions displacing nearby Si atoms, and hence making it easier to adsorb near Al atoms. This result is consistent with the theoretical calculation data: reaction energy barrier for water molecules was smallest in the a region (73.78 kJ mol^−1^), and hence the reaction proceeded easily.

### 4.4 HZSM-5 zeolite reaction path

**Fig. 19:**
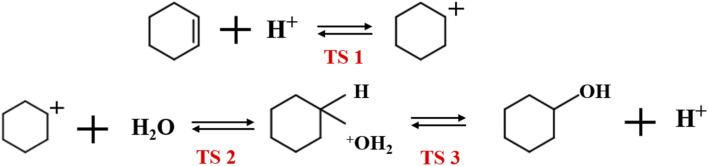
The mechanism of cyclohexene hydration reaction.

Fig. 19 shows the cyclohexene hydration reaction route olefin hydration mechanism can be divided into three stages:

1. The proton on the acidic catalyst was added to the double-bonded carbon atom, generating intermediate carbocation;

2. Intermediate carbocations reacted with water molecules to form protonated alcohols;

3. Protonated alcohol lost protons to form cyclohexanol.

**Fig. 20:**
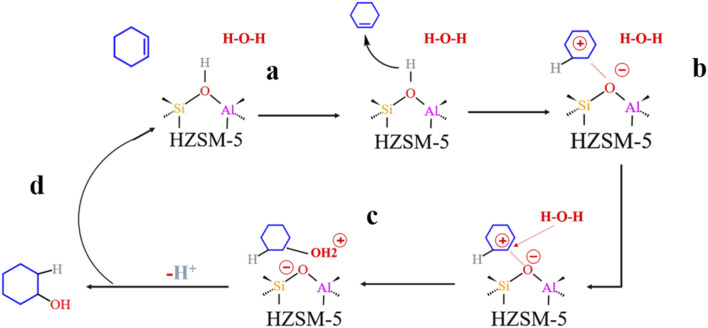
Schematic diagram of the hydration mechanism of cyclohexene catalyzed by HZSM-5.

**Fig. 21:**
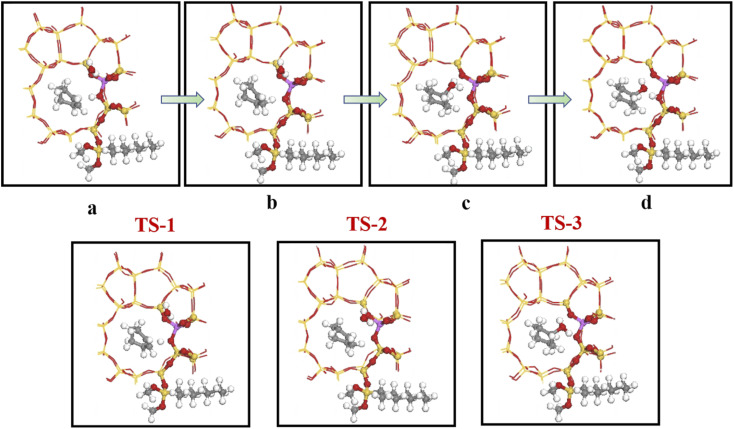
Configuration diagram of adsorption of cyclohexene and water molecules in the pores of HZSM-5 zeolite.

**Fig. 22:**
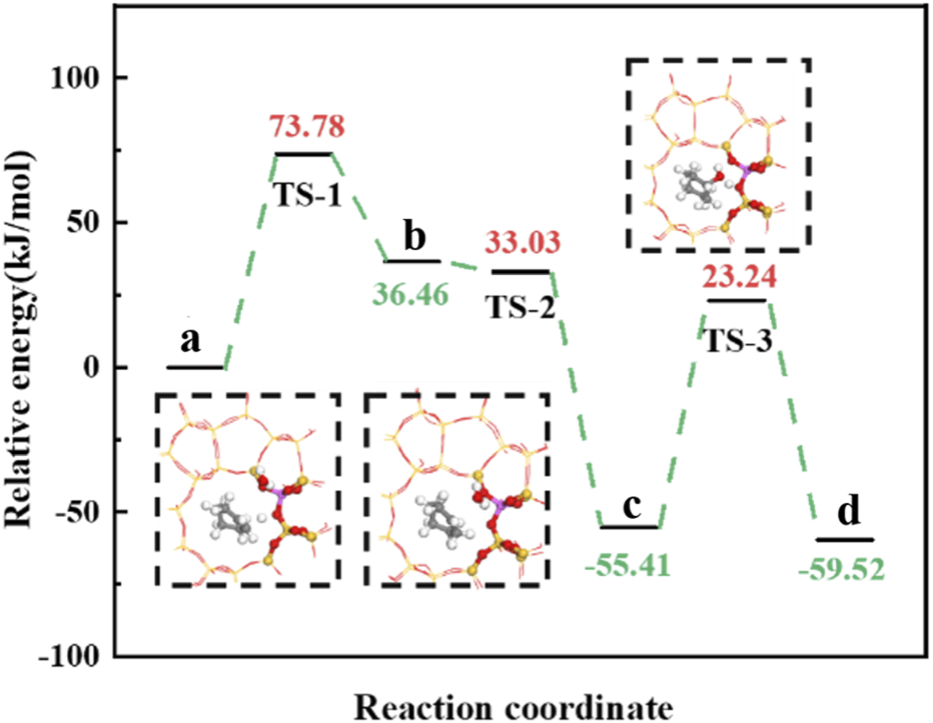
^1–41^HZSM-5 catalyzed cyclohexene hydration energy barrier.

Olefin molecule adsorption on HZSM-5 zeolite generated van der Waals forces within the zeolite framework, the olefin molecule had π electrons, and the B acid centers generated electron induction. Thus, olefin molecules and B acid centers formed a π–H bond. Thus, cyclohexene molecules were protonated by Brønsted acid centers before cleavage reaction, transferring protons to the cyclohexene double-bonded carbon atom. This reaction is consistent with previously observed free fatty acid protonation,^23^ as shown in Fig. 20 and 21. Cyclohexene molecules were protonated by acidic H^+^ to form adsorption intermediates (carbocations), and the TS-1 transition state (Fig. 21), where C_1_–H and C_2_–H distances were transformed to (2.694, 1.968) and (2.085, 1.149) Å, respectively. O_A_–H bond distance extended from 0.977 to 1.769 Å, whereas C_1_–C_2_ bond length increased to (1.331, 1.393) Å. Thus, acidic protons in zeolite moved towards the cyclohexene C_1_–C_2_ bond, forming carbocations (C_1_–H–C_2_). Fig. 22 shows Gibbs activation energy = 73.78 kJ mol^−1^ for cyclohexene protonation from simulation.

The adsorbed carbocation was unstable and highly reactive,^15,16^ and hence can decompose with only small activation energy. Thus it could difficult to locate this intermediate experimentally. Fig. 22 shows that lower activation energies were required for carbocations to generate protonated alcohols and cyclohexanol. Water molecules participated throughout the reaction (Fig. 20(b)–(d)). First, water molecules combined with carbocations to form protonated alcohols with low reaction energy barrier = 33.03 kJ mol^−1^ (Fig. 22). C_1_–H_2_O bond length reduced to 1.529 Å during this process, and C_1_–C_2_ and C_2_–H distances reduced to (1.331, 1.498) and (1.149, 1.085) Å, respectively. Thus, water molecules were adsorbed on the carbocation to form protonated alcohols (Fig. 20(b) and (c)). Protonated alcohols were extremely unstable in the unsaturated cluster model and tended to undergo hydrogen transfer. Experimental results also confirmed activation energy for proton transfer reaction was very small = 23.24 kJ mol^−1^ (Fig. 21, TS-3). C_1_–OH bond length gradually decreased from 1.529 to 1.453 Å, and free protons transferred to the 34T O_A_ atom as O_A_–H bond gradually reduced from 1.632 to 1.033 Å (Fig. 20(c) and (d)). Thus, the proton transfer mechanism can be understood through structural parameter variations.

### 4.5 HZSM-5 zeolite alkylation modification mechanism and reaction path

#### 4.5.1 HCl-HZSM-5 alkylation modification

**Fig. 23:**
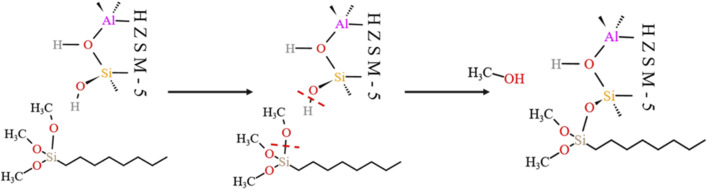
Mechanism of zeolite alkylation modification.

**Fig. 24:**
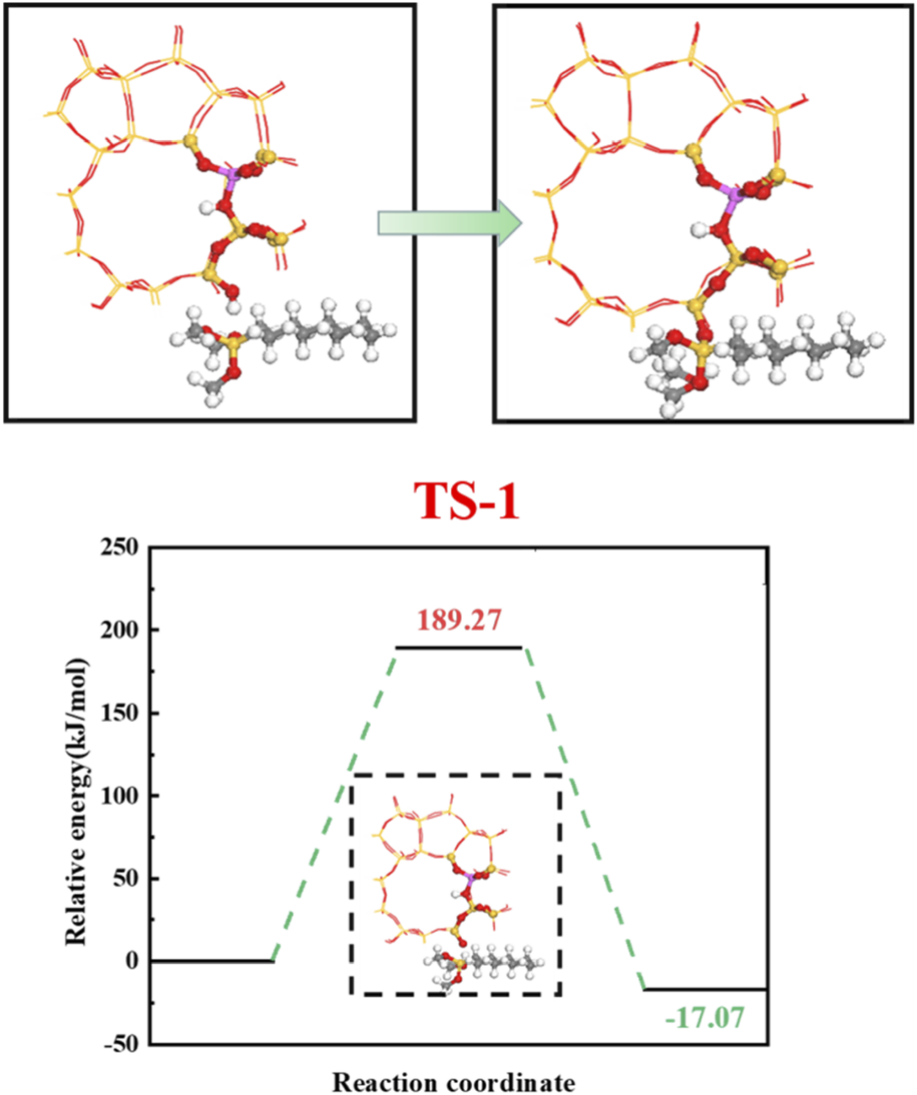
Zeolite alkylation reaction energy barrier.

Fig. 23 shows the zeolite alkylation modification route. The hydroxyl group is cleaved on the surface of the zeolite, then the methyl group (CH_3_–) in *n*-octyltrimethoxysilane (OTS) is cleaved, and the two molecules are subsequently connected by Si–O–Si to complete the alkylation modification, producing HCl-OTS-HZSM-5 zeolite. DPT calculations shows the alkylation modification reaction energy barrier = 189.27 kJ mol^−1^ (Fig. 24).

#### 4.5.2 HCl-OTS-HZSM-5 reaction path

**Fig. 25:**
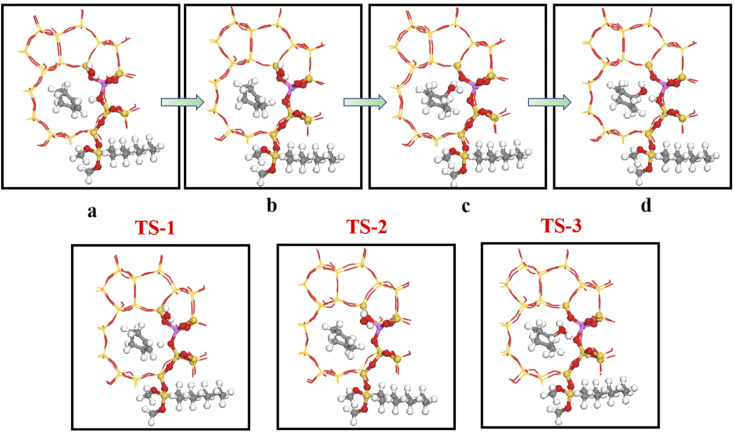
Configuration diagram of adsorption of cyclohexene and water molecules in the pores of HCl-OTS-HZSM-5 zeolite.

**Fig. 26:**
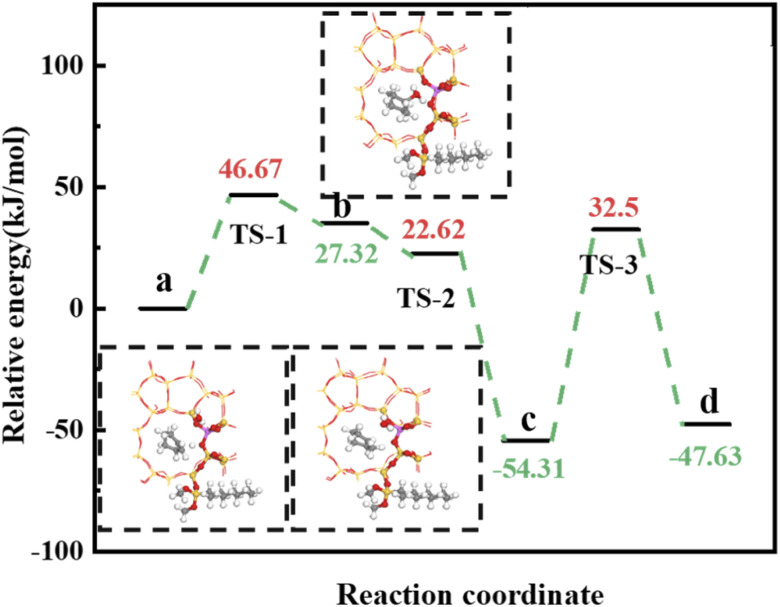
HCl-OTS-HZSM-5 catalyzed cyclohexene hydration energy barrier.

Fig. 25 shows the cyclohexene molecule is protonated by the Brønsted acid center, transferring the proton to the cyclohexene double bond C atom. Adsorbed cyclohexene molecules are protonated by acidic H^+^ to form adsorption intermediates (carbocations) (transition state TS-1, Fig. 25). C_1_–H and C_2_–H bond lengths change to (2.482, 1.947) and (1.871, 1.179) Å, respectively, O_A_–H bond length increases from 1.006 to 1.619 Å, and C_1_–C_2_ bond length increases to (1.334, 1.385) Å. Thus, acidic protons in zeolite moved towards the cyclohexene C_1_–C_2_ bond, forming the C_1_–H–C_2_ carbocation. Fig. 26 shows DPT simulation with reaction energy barrier = 46.47 kJ mol^−1^ (Fig. 26).

Fig. 26 shows adsorbed carbocations combine with water molecules in the zeolite channels to form protonated alcohols (Fig. 25(b) and (c)), with low reaction energy barrier = 22.62 kJ mol^−1^, C_1_–H_2_O bond reduces to 1.556 Å, and C_1_–C_2_ and C_2_–H bond lengths change to (1.385, 1.493) and (1.179, 1.086) Å. Thus, water molecules are adsorbed on the carbocation to form protonated alcohols, which are extremely unstable in the unsaturated cluster model. Dehydrogenation subsequently occurs with Gibbs energy barrier for proton transfer = 32.5 kJ mol^−1^, C_1_–OH bond length gradually decreases from 1.556 to 1.454 Å, and free protons transfer to the HCl-OTS-HZSM-5 zeolite O_A_ atom as O_A_–H bond length reduces to 1.030 Å (Fig. 25(c) and (d)).

Gibbs activation energy for cyclohexene protonation = 73.78 kJ mol^−1^ during unmodified HZSM-5 catalytic reaction (Fig. 21); whereas Gibbs activation energy for protonated cyclohexene = 46.47 kJ mol^−1^ after alkylation modification during the HCl-OTS-HZSM-5 catalytic reaction (Fig. 26). Thus, the reaction energy barrier was greatly reduced after alkylation modification.

**Fig. 27:**
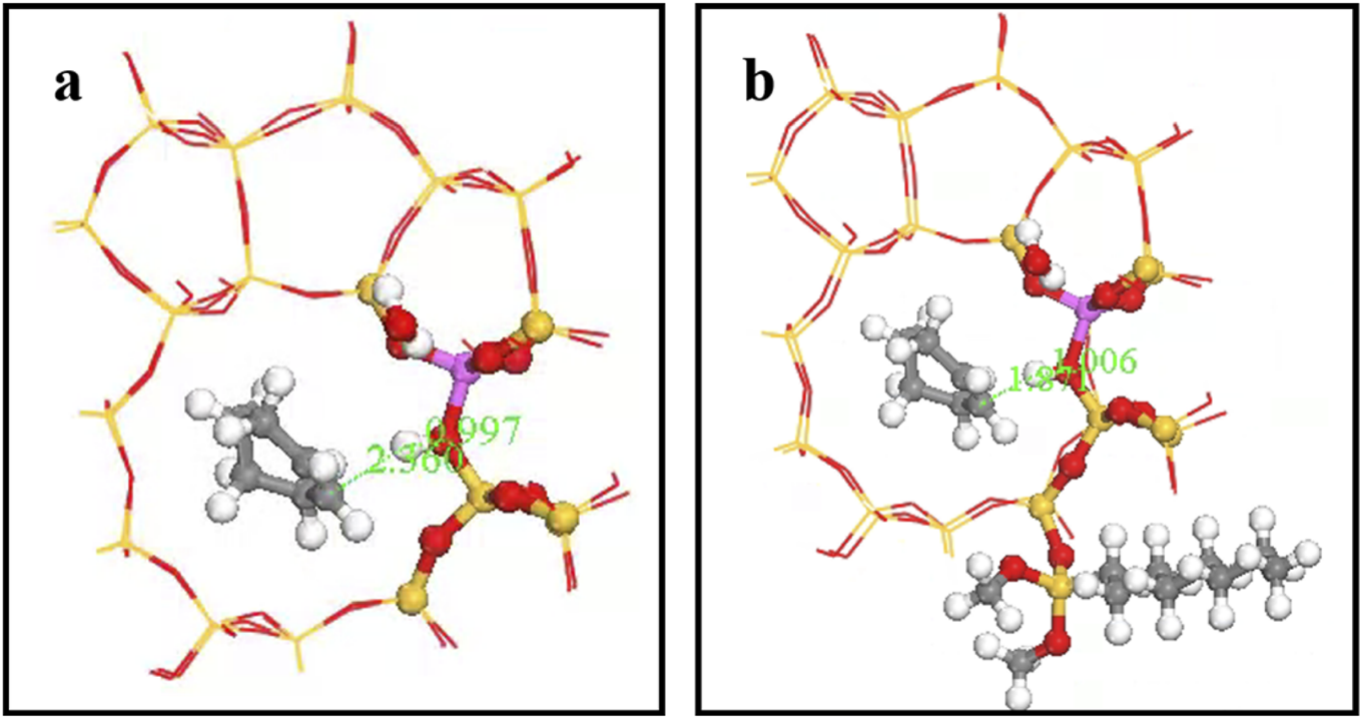
O_A_–H bond length after adsorption of HZSM-5 and HCl-OTS-HZSM-5.

Fig. 27 shows O_A_–H bond lengths = 0.997 and 1.006 Å; whereas C_2_–H bond length = 2.360 and 1.871 Å before and after adsorption, respectively. Thus, two-phase reactant contact was macroscopically enhanced after alkylation modification. Microscopically, water molecules were more likely to activate hydrogen protons, increasing O_A_–H bond length and B acid strength, and hence reducing the reaction energy barrier to more easily form carbocations. This is consistent with experimental outcomes, and hence the reaction conversion rate was greatly improved after alkylation modification.

## 5 Conclusion

This paper prepared HCl-OTS-HZSM-5 zeolite catalyst by acid and alkylation modification and investigated olefin hydration reaction effects. Acid modification removes some HZSM-5 zeolite aluminum atoms, increasing specific surface area, pore volume, and acidic active sites. Acid-modified catalyst exhibited good hydrothermal stability and configurational stability for hydrothermal processes, enhancing cyclohexene hydration reactions. We grafted *n*-octyltrimethoxysilane (OTS) onto the HZSM-5 catalyst surface to improve interfacial mass transfer limitations for the two-phase reaction, forming Pickering emulsion which improved contact between immiscible reactants. Consequently, cyclohexene conversion reached 24.07%, with relatively stable selectivity close to 100%.

We constructed a 34T cluster model using the mGGA-M06-L function for DFT calculations to represent the zeolite channel structure, and investigated reaction activation energy for cyclohexene hydration in HZSM-5 and HCl-OTS-HZSM-5 zeolite channels. Simulations confirmed that bridging hydroxyl groups preferentially protonated alkenes at low water loadings, and water molecules were more easily adsorbed near less shielded Al atoms. The highest reaction energy barrier for HCl-OTS-HZSM-5 zeolite catalyzed by cyclohexene hydration reaction was only 46.67 kJ mol^−1^, which greatly reduced the reaction energy barrier. Simulations also confirmed alkylation modification feasibility.

## Conflicts of interest

There are no conflicts to declare.

## Supplementary Material

RA-012-D2RA04285A-s001
