# Supplementary material for: HZSM-5 zeolite modification and catalytic reaction mechanism in the reaction of cyclohexene hydration
Source: RSC Adv. 2022 Aug 31;12(38):24654–69. doi: 10.1039/d2ra04285a (PMC9429031; doi:10.1039/d2ra04285a)
Supplement: RA-012-D2RA04285A-s001 [file RA-012-D2RA04285A-s001.pdf]

Electronic Supplementary Material (ESI) for RSC Advances.
This journal is © The Royal Society of Chemistry 2022

Calculation method

DFT calculations were performed using the $DMOL_3$ module in Materials Studio 2017 software, employing the hyper generalized approximate gradient (m-GGA) with M60-L for all atoms [31]. The m-GGA density functional offered thermodynamic, kinetic, non-covalent interaction, bond length, reaction energy barrier, and vibrational frequency advantages. We adopted dual-value polarization basis set polarization function to consider electron polarization effects. All electronic calculations were performed on the complex domain with unrestricted system spin state, i.e., SCF = 1.0×10^{-5}, maximum SCF cycles = 800, and maximum iterations = 800.

We used a full LST/QST to search for transition states and hence determine the activation energy for a specific path. Only one imaginary frequency corresponds to the expected atomic motion in each transition structure. Convergence thresholds for energy, gradient, and displacement were 2.0×10^{-5} Ha, 0.004 Ha/A, and 0.005 A, respectively. Eyring [32] proposed the transition state theory for chemical reactions due to elementary reaction (Eα) activation energy could be expressed as:

$$\Delta E=E(P)-EI \tag{1}$$

$$E\alpha=E(TS)-EI \tag{2}$$

Where EI, E(TS), and E(P) are electronic ground state energies for the reactant, transition, and product state, respectively. All energies were corrected by zero-point vibrational energy and Gibbs free energy.

Material characterization

Analyses were performed using X-ray diffraction (XRD), Shimadzu Corporation XRD 6000, Using Cu target (Ka ray, λ = 1.4518 Å), Ni filter, working condition of 40 kV, working current of 30 mA, scanning within 2θ=10°~80°, and scanning speed of 20°/min; Fourier Transform Infrared Spectroscopy (FT-IR), Thermo Corporation of America Nicolet-380, Using KBr as the background, according to the ratio of catalyst to KBr of 1:200, the tablet was pressed at a pressure of 1.75 Mpa for 1 min, and the sample was scanned after the tablet was pressed.; Temperature-programmed desorption

analysis of NH_3 (NH_3-TPD), American Micromeritics Auto Chem II 2920, The samples were replaced in nitrogen at 200 °C for 1 h, and then NH_3 was used for adsorption. Nitrogen gas was introduced into the reactor for 20 min, and then the TPD curve was recorded by increasing the temperature from 30 °C to 700 °C at a rate of 10 °C/min; Pyridine adsorption infrared spectroscopy (Py-IR), Germany Bruker Optics Tensor 27, The samples were pretreated at high temperature and high vacuum for 2 hours before pyridine adsorption, and then vacuum heated in situ; Scanning Electron Microscopy Analysis (SEM), Nippon Electronics Co., Ltd. JSM-7900F, The sample powders were modified by gold spraying and then characterized by scanning electron microscopy operating at 1.0 kV; Specific surface area and pore volume and pore size analysis (BET), American Micromeritics ASAP2020, The sample was first evacuated, and then high-purity nitrogen was used as the adsorbate, and the liquid nitrogen was adsorbed at low temperature to determine the specific surface area and pore volume of the sample; Contact angle (CA), German Dataphysics JY-82B Kruss DSA, The samples were compressed to a thickness of 2 mm and placed in a 1 cm disc (OD) and characterization determinations were made using optical contact angle measurements and ink droplet profiles; Thermogravimetric-Differential Thermal Analysis (TGA), Germany Netzsch STA 449C, Weigh 8 mg of sample, use high-purity nitrogen as protective gas, increase the temperature from 25 °C to 800 °C at a heating rate of 10 °C/min, and record the weight loss of the sample; X-ray photoelectron spectroscopy (XPS), Thermo Corporation of America Scientific K-Alpha to confirm successful acid treatment and OTS grafting and subsequent effects. We subsequently evaluated HCl-OTS-HZSM-5 effects for cyclohexene hydration under various conditions.

Table information

Table. S1 Number of Mulliken electron layouts and distances between atoms before and after cyclohexene adsorption

Cyclohexene atomic distribution	Mulliken electron layout number (e)		Cyclohexene atomic distribution	Atomic distance (Å)	
	Before adsorption	After adsorption		Before adsorption	After adsorption
C_1	-0.132	-0.089	C_1-C_2	1.331	1.498
C_2	-0.131	-0.293	C_2-C_3	1.494	1.504
C_3	-0.385	-0.54	C_3-C_4	1.523	1.502
C_4	-0.348	-0.453	C_4-C_5	1.520	1.523
C_5	-0.347	-0.503	C_5-C_6	1.523	1.484
C_6	-0.385	-0.528	C_6-C_1	1.494	1.333
H_{C1}	0.158	0.220	C_1-H	1.088	1.079
H_{C2}	0.158	0.274	C_2-H	1.088	1.099
H_{C31}	0.181	0.234	C_3-H	1.097	1.083
H_{C32}	0.181	0.259	C_4-H	1.098	1.090
H_{C41}	0.172	0.213	C_5-H	1.095	1.087
H_{C42}	0.172	0.258	C_6-H	1.101	1.079
H_{C51}	0.172	0.239			
H_{C52}	0.172	0.247			
H_{C61}	0.181	0.247			
H_{C62}	0.181	0.300			
SUM	0	0.085			

Table. S2 The number of Mulliken electron layouts and the distance between atoms before and after the adsorption of 34T-HZSM-5

34T-HZSM-5 atomic distribution	Mulliken electron layout number (e)	
	Before adsorption	After adsorption
H	0.568	0.616
O_A	-1.192	-1.237

34T-HZSM-5 atomic distribution	Atomic distance (Å)	
	Before adsorption	After adsorption
O_A-H	0.978	0.997
Al-O_A	1.828	1.816
Si-O_A	1.673	1.670
